# Effects of the reservoir bag disconnection on inspired gases during general anesthesia: a simulator-based study

**DOI:** 10.1186/s12871-021-01256-2

**Published:** 2021-02-01

**Authors:** Miljenko Križmarić, Uroš Maver, Marko Zdravković, Dušan Mekiš

**Affiliations:** 1 Faculty of Medicine, University of Maribor, Maribor, Slovenia; 2 Faculty of Health Sciences, University of Maribor, Maribor, Slovenia; 3 Department of Anesthesiology, Intensive Care and Pain Management, University Medical Centre Maribor, Maribor, Slovenia

**Keywords:** Equipment design, Anesthesia ventilators, Ventilation, Low-flow anesthesia, Fresh gas decoupling, Patient safety

## Abstract

**Background:**

Fresh gas decoupling is a feature of the modern anesthesia workstation, where the fresh gas flow (FGF) is diverted into the reservoir bag and is not added to the delivered tidal volume, which thus remains constant. The present study aimed to investigate the entraining of the atmospheric air into the anesthesia breathing circuit in case the reservoir bag was disconnected.

**Methods:**

We conducted a simulator-based study, where the METI HPS simulator was connected to the anesthesia workstation. The effect of the disconnected reservoir bag was evaluated using oxygen (O_2_) and air or oxygen and nitrous oxide (N_2_O) as a carrier gas at different FGF rates. We disconnected the reservoir bag for 10 min during the maintenance phase. We recorded values for inspiratory O_2_, N_2_O, and sevoflurane. The time constant of the exponential process was estimated during reservoir bag disconnection.

**Results:**

The difference of O_2_, N_2_O and sevoflurane concentrations, before, during, and after reservoir bag disconnection was statistically significant at 0.5, 1, and 2 L/min of FGF (*p* < 0.001). The largest decrease of the inspired O_2_ concentrations (F_I_O_2_) was detected in the case of oxygen and air as the carrier gas and an FGF of 1 L/min, when oxygen decreased from median [25th–75th percentile] 55.00% [54.00–56.00] to median 39.50% [38.00–42.50] (*p* < 0.001). The time constant for F_I_O_2_ during reservoir bag disconnection in oxygen and air as the carrier gas, were median 2.5, 2.5, and 1.5 min in FGF of 0.5, 1.0, and 2 L/min respectively.

**Conclusions:**

During the disconnection of the anesthesia reservoir bag, the process of pharmacokinetics takes place faster compared to the wash-in and wash-out pharmacokinetic properties in the circle breathing system. The time constant was affected by the FGF rate, as well as the gradient of anesthetic gases between the anesthesia circle system and atmospheric air.

## Background

In conventional anesthesia workstations, fresh gas flow (FGF) is delivered continuously from the anesthesia machine into the anesthesia circle breathing system, regardless of the phase of mechanical ventilation. The fraction of FGF, which is added during the inspiratory phase, increases the delivered tidal volume (V_T_) and can potentially cause volutrauma and/or barotrauma [1–3]. Such FGF set up is called fresh gas coupling. Ventilators prone to the latter are especially unsuitable for neonates due to the unpredictability of the delivered V_T_ [4].

With technological developments, the anesthesia workstation and the circle breathing systems have evolved to include fresh gas decoupling (FGD) systems thus improving patient safety. In FGD systems, a decoupling valve diverts the FGF toward the reservoir bag during the inspiratory phase of controlled mechanical ventilation. The FGF is thus not directed to the volume delivered by the ventilator during the inspiratory phase of ventilation. By varying the FGF, respiratory rate and/or I:E ratio no changes in delivered V_T_ were noted in FGD systems [5, 6].

The main disadvantage of ventilators that use an FGD system is the possibility of entraining atmospheric air into the anesthesia breathing circuit if the reservoir bag was accidentally disconnected resulting in patient awareness or hypoxia [7]. For ethical reasons, such scenarios are challenging to be trialed in a real clinical environment. Advanced simulators, however, such as the Human Patient Simulator (HPS), enable physiologically authentic replication of various clinical situations [8]. For example, the HPS is designed to consume oxygen and anesthetic gases, exhale CO_2_ and is programmed to respond even to delivered drugs, such as sedatives, opioids, and neuromuscular blocking drugs. Additionally, when the anesthetic workstation is connected to the HPS manikin, the concentrations of delivered gases are set on the vaporizer and are easily measured using the same technology as in daily clinical practice [9].

The aim of this simulator-based study was to determine the changes in inspired gas concentrations when the reservoir bag was intentionally disconnected from the anesthesia circle breathing system. The obtained data is then used to discuss potential clinical implications of such scenarios and how to overcome them to the benefit of patients.

## Methods

**Fig. 1:**
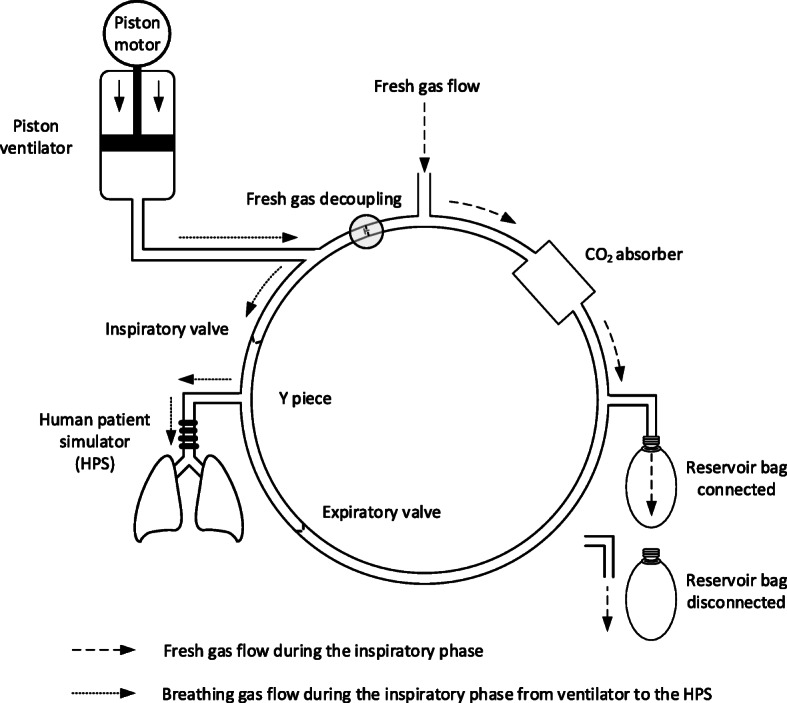
Simplified gas flow diagram in inspiratory phase of Dräger Primus workstation with an electrically driven piston ventilator and fresh gas decoupling [10]

This simulator-based study took place at the Simulation Centre of Faculty of Medicine, University of Maribor, Slovenia. We used HPS (Human Patient Simulator METI, Sarasota, FL) connected to the Dräger Primus anesthesia workstation (Drägerwerk AG & Co. KGaA, Lübeck, Germany) through a circle breathing system to simulate a reservoir bag disconnection during general anesthesia. The Dräger Primus workstation incorporates an electrically driven piston ventilator and fresh gas decoupling (FGD) system (Fig. 1) [10].

Before the experiments, the anesthesia workstation was tested with the complete automated self-test in accordance with the manufacturer’s instructions. Inspiratory concentrations of the anesthetic gases were monitored on the anesthesia workstation gas monitor. The gas sample line was connected to an anesthesia circle system with an internal volume of 1.2 L (breathing circuit 1.6 m, Intersurgical. Workingham, UK).

In the simulation experiments the HPS manikin represented a 70 kg adult male patient undergoing general anesthesia. HPS was preoxygenated with 100% O_2_ at FGF of 6 L/min for 3 min and intubated with an 8.0 mm tracheal tube. After tracheal intubation, positive pressure ventilation was initiated with a V_T_ of 500 mL, a respiratory rate of 12 breaths/min, I:E ratio of 1:2, ratio of inspiratory pause time to inspiration time of 10%, and PEEP of 0 cm H_2_O (the manufacturer recommends 0 PEEP to protect the manikin). Muscle relaxation was set at 100% throughout the experiments.

Following tracheal intubation two different simulation scenarios were run involving different anesthetic carrier gases: either oxygen and air or oxygen and nitrous oxide. When oxygen and air was the carrier gas, sevoflurane of 3.5 vol% and 40 O_2_ was delivered to the circle breathing system at the FGF of 4 L/min (initial phase of general anesthesia). In the maintenance phase, we adjusted the delivered O_2_ concentration to 68% and sevoflurane to 5 vol% at three different FGFs: 0.5 L/min, 1.0 L/min, and 2.0 L/min. In the second simulation scenario using oxygen and nitrous oxide as the carrier gas, the initial phase included the delivery of 32% O_2_ and 2 vol% sevoflurane at the FGF of 4.4 L/min. In the maintenance phase, oxygen was set to 60% and sevoflurane to 3.5 vol% at three different FGFs: 0.5 L/min, 1.0 L/min, and 2.0 L/min [11]. The above protocol is generally only proposed for the minimal flow inhalational anesthesia (0.5 L/min). However, we have used the same procedure also for higher flow rates (1.0 and 2.0 L/min) to obtain more dispersed values for inspired gases just before reservoir bag disconnection to show the effects of the latter more clearly.

To keep a consistent time scale, as well as to allow for an exact comparison of the respective runs, all simulation runs were set up to have the same time duration for different sequential phases: (1) atmospheric (room) air breathing for 5 min, (2) preoxygenation for 3 min, (3) initial phase for 10 min, (4) maintenance phase for 20 min, (5) disconnected reservoir bag for 10 min, and (6) reconnected reservoir bag for 20 min. Each simulation experiment was repeated 3 times for each FGF.

Statistical analysis was performed using IBM SPSS Statistics 26 (IBM Corp., Armonk, N.Y., USA). The data are presented as median [25th–75th percentile]. *P*-values < 0.05 were considered statistically significant. Gas concentrations 10 min before to, 10 min during, and 10 min after reservoir bag disconnection were analyzed using the Fridman nonparametric test. The time constant (*τ*) is the time, after anesthetic concentration in the breathing system will attain 63% of its final value. To determine its final value, we determined the minimum concentration value at the end of the 10-min interval during reservoir bag disconnection, which was used as the basis for the calculation of this time constant.

## Results

In the tables, we present the statistical values obtained from the three experiments. In each experiment, the values were recorded at 10-min intervals. In total, we have 30 measurements before and during the disconnection of the reservoir bag and 30 measurements during the reservoir bag reconnection phase. On the other hand, the figures show representative examples of time courses of variables from one experiment throughout the experiment’s time window (room air-breathing, preoxygenation, initial phase, maintenance phase, disconnected reservoir bag, and reconnected reservoir bag.

### Oxygen and air as the carrier gas

**Fig. 2:**
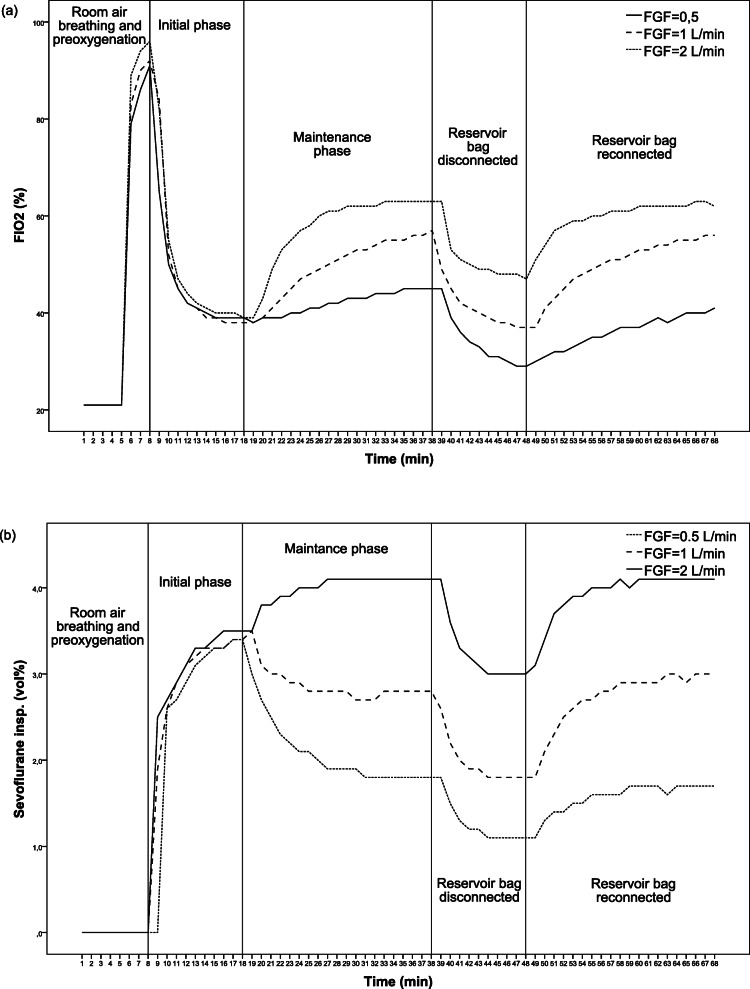
Inspired fraction of oxygen (**a**) and sevoflurane (**b**) curves at variable rate of fresh gas flow (FGF) and oxygen and air as the carrier gas

In the first part of the study, the effect of the reservoir bag disconnection was evaluated using oxygen and air as the carrier gas. Inspired O_2_ and sevoflurane concentrations 10 min before to reservoir bag disconnection, 10 min during reservoir bag disconnection, and 10 min during the reservoir bag reconnection are presented in Table 1. The differences in inspired concentrations of oxygen as well as the differences in sevoflurane concentrations between these three ten minutes time intervals were statistically significant at all FGFs tested (*p* < 0.001, for all). Typical time courses of the inspired fraction of oxygen and sevoflurane at 0.5, 1, and 2 L/min of FGF are presented in Fig. 2.

**Table 1:** Inspired fraction of oxygen and sevoflurane before, during and after the reservoir bag was disconnected in the case of oxygen and air as the carrier gas

Inspired gas	FGF	Inspired fraction before disconnection of reservoir bag (%)	Inspired fraction during disconnection of reservoir bag (%)	Inspired fraction during reconnection of reservoir bag (%)	P^*^
Oxygen	0.5	44.00 [41.75–45.00] Min = 40.00 Max = 46.00	31.50 [30.00–35.25] Min = 28.00 Max = 45.00	33.00 [31.75–34.25] Min = 29.00 Max =37.00	< 0.001
	1.0	55.00 [54.00–56.00] Min = 52.00 Max = 58.00	39.50 [38.00–42.50] Min = 37.00 Max = 49.00	48.00 [43.75–50.00] Min = 37.00 Max = 52.00	< 0.001
	2.0	64.00 [63.00–64.00] Min = 62.00 Max = 65.00	50.00 [49.00–52.00] Min = 47.00 Max = 64.00	60.00 [57.50–61.00] Min = 51.00 Max = 63.00	< 0.001
Sevoflurane	0.5	1.90 [1.80–1.90] Min = 1.80 Max = 2.00	1.2 [1.10–1.35] Min = 1.10 Max = 1.80	1.50 [1.40–1.60] min = 1.10 Max = 1.70	< 0.001
	1.0	2.90 [2.80–2.90] Min = 2.70 Max = 3.00	1.90 [1.90–2.20] Min = 1.80 Max = 2.70	2.70 [2.37–2.80] Min = 1.80 Max = 3.00	< 0.001
	2.0	4.10 [4.00–4.10] Min = 4.00 Max = 4.10	3.10 [3.10–3.40] Min = 3.00 Max = 4.10	3.90 [3.68–4.00] Min = 3.10 Max = 4.10	< 0.001

^*^Friedman nonparametric test. Values are expressed as median [25th–75th percentile]

*Min* minimum, *Max* Maximum, *FGF* fresh gas flow (L/min)

### Oxygen and nitrous oxide as the carrier gas

**Fig. 3:**
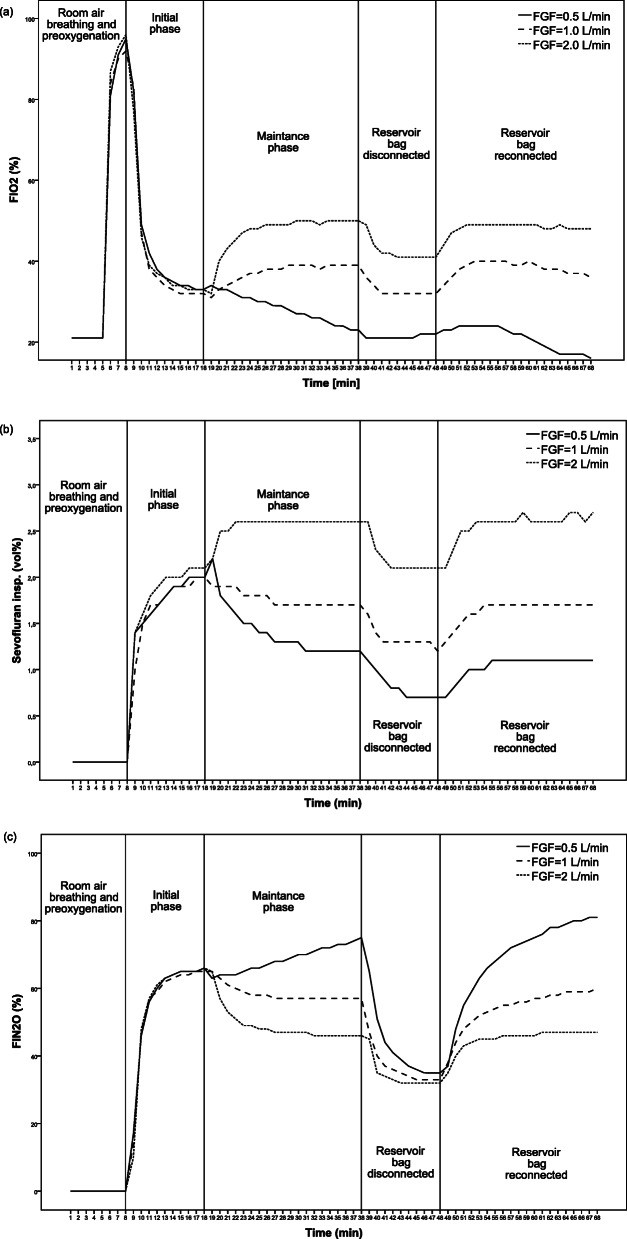
Inspired fraction of oxygen (**a**), sevoflurane (**b**) and nitrous oxide (**c**) curves at the variable rate of fresh gas flow (FGF) and oxygen and nitrous oxide as the carrier gas

In the second part of the study, the oxygen and nitrous oxide was used as a carrier gas. There were significant differences of anesthetic gases detectable at 0.5, 1, and 2 L/min of FGF between 10 min before to reservoir bag disconnection, 10 min during the reservoir bag disconnection and 10 min during reservoir bag reconnection (*p* < 0.001, for all) (Table 2). Typical time courses of inspired fraction of oxygen, sevoflurane and nitrous oxide at 0.5, 1, and 2 L/min of FGF are presented in Fig. 3.

**Table 2:** Inspired fraction of oxygen before, during and after the reservoir bag was disconnected in the case of oxygen and nitrous oxide as the carrier gas

Inspired gas	FGF	Inspired fraction before disconnection of reservoir bag (%)	Inspired fraction during disconnection of reservoir bag (%)	Inspired fraction during reconnection of reservoir bag (%)	p*
Oxygen	0.5	26.00 [24.00–27.00] Min = 22.00 Max = 29.00	21.00 [20.00–21.50] Min = 19.00 Max = 22.00	23.00 [20.00–24.00] Min = 18.00 Max = 24.00	< 0.001
	1.0	39.00 [38.00–39.00] Min = 38.00 Max = 40.00	32.00 [31.00–32.00] Min = 32.00 Max = 36.00	37.50 [36.00–39.00] Min = 33.00 Max = 40.00	< 0.001
	2.0	50.00 [49.00–50.00] Min = 49.00 Max = 51.00	42.00 [41.00–43.00] Min = 41.00 Max = 49.00	49.00 [48.00–49.25] Min = 43.00 Max = 51.00	< 0.001
Sevoflurane	0.5	1.20 [1.20–1.20] Min = 1.20 Max = 1.30	0.80 [0.70–0.90] Min = 0.70 Max = 1.10	1.00 [0.90–1.10] Min = 0.70 Max = 1.10	< 0.001
	1.0	1.90 [1.70–1.90] Min = 1.70 Max = 1.90	1.30 [1.30–1.40] Min = 1.20 Max = 1.80	1.70 [1.60–1.80] Min = 1.30 Max = 1.90	< 0.001
	2.0	2.60 [2.60–2.70] Min = 2.60 Max = 2.70	2.20 [2.10–2.30] Min = 2.10 Max = 2.60	2.60 [2.47–2.60] Min = 2.10 Max = 2.80	< 0.001
Nitrous oxide	0.5	71.00 [69.00–72.25] Min = 67.00 Max = 75.00	38.00 [36.00–43.25] Min = 35.00 Max = 65.00	65.50 [55.75–72.00] Min = 37.00 Max = 76.00	< 0.001
	1.0	57.00 [57.00–58.00] Min = 57.00 Max = 59.00	33.50 [33.00–36.25] Min = 32.00 Max = 47.00	53.00 [48.00–55.00] Min = 38.00 Max = 57.00	< 0.001
	2.0	46.00 [46.00–46.00] Min = 45.00 Max = 47.00	33.00 [32.00–34.00] Min = 32.00 Max = 45.00	45.00 [43.00–45.00] Min = 35.00 Max = 46.00	< 0.001

*Friedman nonparametric test. Values are expressed as median [25th–75th percentile]

*Min* minimum, *Max* Maximum, *FGF* fresh gas flow (L/min)

Time constants of anesthetic gases during 10 min of the reservoir bag disconnection are presented in Table 3.

**Table 3:** Time constants for inspired concentrations od oxygen, sevoflurane and nitrous oxide, during reservoir bag disconnection at variable rates of fresh gas flows (FGF) and different carrier gases

FGF (L/min)	Time constant (minutes) Oxgen and air as the carrier gas		Time constant (minutes) Oxgen and nitrous oxide as the carrier gas		
	F_I_O_2_	F_I_Sevo	F_I_O_2_	F_I_Sevo	F_I_N_2_O
0.5	2.5 [2.5–2.5]	2.0 [1.8–2.25]	1.0 [1.0–1.0]	3.0 [2.3–3.0]	1.5 [1.5–1.8]
1.0	2.5 [2.5–2.8]	2.0 [1.8–2.0]	1.0 [1.0–1.3]	2.0 [1.8–2.0]	1.5 [1.3–1.5]
2.0	1.5 [1.5–1.5]	1.5 [1.5–1.8]	1.0 [1.0–1.0]	1.0 [1.0–1.5]	1.0 [1.0–1.0]

Values of the time constant are expressed as median [25th–75th percentile]

*FGF* fresh gas flow, *F*_*I*_*N*_*2*_*O* inspired nitrous oxide concentration, *F*_*I*_*O*_*2*_ inspired O_2_ concentration, *F*_*I*_*Sevo* inspired sevoflurane concentration

## Discussion

This simulator-based study demonstrates that a reservoir bag disconnection leads to a significant decrease of inspired anesthetic gases and oxygen resulting from the entrainment of atmospheric (room) air due to a large leak in the circle breathing system.

The values for the delivered gas fraction and the procedures chosen in the study were determined by the guidelines for minimum flow inhalation anesthesia performed at an FGF of 0.5 L / min. The experiments performed are consistent with those reported in these references [11–14]. The same values of delivered gas fraction and fresh gas flows were used for other performed experiments (at FGF of 1.0 and 2.0 L/min) since different inspiratory oxygen values were obtained just before the breathing bag disconnection. Different clinical situations (e.g., pathophysiology changes in the respiratory system) or problems involved the anaesthesia workstation/equipment (e.g., Intraoperative anesthesia equipment failures) can lead to different F_I_O_2_ values during general anesthesia. The study’s main focus was on the time course of oxygen change because of potential patient hypoxia in case of insufficient oxygen supply. The administration of drugs to the simulator during anesthesia induction and in later stages was not necessary, as the HPS simulator for such cases has an automatic adjustment option (i.e., muscle relaxation – 100%).

We found that the largest decrease of the inspired oxygen concentrations was detected at oxygen and air as the carrier gas and FGF of 1 L/min, when oxygen decreased from a median 55.00 to a median 39.50%. This process follows the circuit-to-atmospheric air oxygen concentration gradient (55–21% of O_2_). During the 10 min when the reservoir bag was disconnected and using oxygen and air as the carrier gas, the inspired oxygen concentrations were decreasing from maximum to minimum: 45–28, 49–37, and 64–47% by FGF of 0.5, 1, and 2 L/min respectively. With oxygen and nitrous oxide as the carrier gas, inspired oxygen concentrations decreased, as follows 22–19, 36–32, and 49–41%. There are a few case reports in the literature describing such conditions. Sandberg and Kaiser [15] describe the entrainment of room air through a hole in a reservoir bag. In the clinical case described, initial settings on Dräger Fabius GS workstations were 2 L/min of nitrous oxide, 1 L/min of oxygen (oxygen delivery of 33%), and 2 vol% desflurane. After a few minutes, they noted an empty reservoir bag and recorded a fall to 23% oxygen, 52% nitrous oxide, and 1.3% desflurane. Roiss [16] et all report of an intraoperative drop in sevoflurane and oxygen concentration that occurred during low flow anesthesia on a Dräger Primus workstation. In this second clinical case, delivered oxygen was set to 55% and FGF to 0.8 L/min. They noticed that the disconnection of the reservoir bag could be identified as the cause of the drop in gas concentrations (F_I_O_2_ from 36 to 29%, and sevoflurane from 2.0 to 1.3 vol%. The reported percentage drops from initial values in this clinical setting were comparable to the ones observed in our study (F_I_O_2_ drop from 45 to 28%; FGF = 0.5 L/min; Table 1), although the observed absolute % drop in our study was even more prominent. Problems with gas leakage in a faulty CO_2_ absorber are similar to a reservoir bag disconnection. In the inspiration phase, the gas flows from FGF through the absorber and then into the reservoir bag (as shown in Fig. 1). Vinay et al. [17] reported that disconnecting a refillable CO_2_ absorber leads to a progressive fall of both the inspired oxygen and the anesthetic agent concentration. FGF initial value of the oxygen was 2 L/min O_2_ and nitrous oxide was 2 L/min (oxygen delivery of 50%). During the operating procedure, they disconnected the absorber to change soda lime after increasing fresh gas flow to 6 L/min. After the disconnection value of F_I_O_2_ decreased from 50 to 31%. In our study, the observed drop was from 49 to 41%, although it has to be noted that we used a lower FGF (FGF = 2 L/min, Table 2).

The internal volume of the Dräger Primus anesthesia workstation in automatic ventilation without breathing hoses was approximately 4.7 L (including piston volume and refillable CO_2_ absorber) [18]. Instead of a refillable CO_2_ absorber with a volume of 1.5 L, we used the Dräger CLIC absorber with a slightly smaller volume (1.2 L). The total gas volume (V_tot_) with a breathing circle volume of 1.2 L was, therefore, 5.6 L (4.7–1.5 + 1.2 + 1.2 L).

The dynamics of wash-in and wash-out of the breathing circuit are time (*t*) dependant. Circuit fraction (F_circ_) of gas approaches delivered fraction (F_del_) following an exponential time course: ${F}_{circ}(t)={F}_{circ}(0)+\left({F}_{del}-{F}_{circ}(0)\right)\bullet \left(1-{e}^{-\frac{t}{\tau }}\right)$, with a time constant *τ* = *V*_*tot*_/*FGF* [19]. In our study, theoretically calculated time constants at variable FGF rate of 0.5, 1, and 2 L/min were 11.2, 5.6, and 2.8 min, respectively. When we disconnected the reservoir bag, the time constants for F_I_O_2_ in oxygen and air as the carrier gas, were mean 2.5, 2.5, and 1.5 min in FGF of 0.5, 1.0, and 2 L/min, respectively. Based on our calculations, as well as the clinical observations in the cases mentioned above, it can be stated that these processes are much faster compared to the wash-in and wash-out kinetics of the circuit. In the case of oxygen and nitrous oxide as the the carrier gas, the same time constant for F_I_O_2_ of mean 1 min was determined for all FGFs. Hence, if the reservoir bag is absent, the delivery of a lower than expected oxygen concentration could occur. Unlike the traditional circle system, in a fresh gas decoupling circuit, the reservoir bag is always in the circuit whether ventilation is spontaneous or controlled [15]. However, the anesthesia reservoir bag, which is normally quiescent during mechanical ventilation, moves with each ventilation cycle in the fresh gas decoupling circuit providing the function of a “visual monitor”. Fresh gas decoupling systems are integrated with the following anesthesia workstations: Dräger Fabius GS, Dräger Narkomed 6000, Dräger Narkomed 6400, Dräger Primus, Dräger Apollo, and Datascope Anestar. Modern anesthesia workstations without FGD, such as Dräger Zeus and Perseus, incorporate turbine-type ventilators where a turbine blower generates flow and pressure directed into the inspiratory limb of the patient circuit, drawing gas from the reservoir bag, which serves as a reservoir during mechanical ventilation. As in the piston ventilators with FGD, the reservoir bag is an integral part of the circuit during mechanical ventilation, continuing to serve a reservoir function [5]. If the reservoir bag is removed during mechanical ventilation, or if it has a significant leak either due to to bag mount disconnection or a perforation, room air may enter the breathing circuit. This may result in dilution of the inhaled anesthetic agents, the enriched oxygen mixture, or both. Furthermore, this type of disruption could lead to signifcant contamination of the operating room atmosphere with anesthetic gases as fresh gases would be allowed to escape into the atmosphere. However, if this occurs, an alarm will alert the operator. If unnoticed, this dilution of patient gases could lead to intraoperative patient awareness or hypoxia [20].

The dynamics of O_2_ are slightly different at FGF of 0.5 L/min, and O_2_ and air as the carrier gas, where inspired oxygen almost linearly increases throughout the entire maintenance phase (Fig. 2). While using the same FGF and oxygen and nitrous oxide as the carrier gas there were minor differences and inspired oxygen falls throughout the maintenance phase (Fig. 3). The oxygen delivery was 340 mL O_2_/min (68% out of 0.5 L/min) in the case of oxygen and air as the carrier gas, and 300 mL O_2_/min (60% out of 0.5 L/min) in the case of oxygen and nitrous oxide as the carrier gas. We can estimate an oxygen consumption of the METI simulator between 300 and 340 mL/min despite setting it to 250 mL/min. According to the Conway [21] equation, the time constant (*τ*) is proportional to the volume of the system including the anesthesia breathing circuit and the lungs (*V*_*S*_); and inversely proportional to the difference between the amount of anesthetic agent delivered into the breathing system ($\dot{V_D}$) at the time and at a constant rate of uptake (${\dot{V}}_{uptake}$): $\tau ={V}_S/\left(\dot{V_D}-{\dot{V}}_{uptake}\right)$. To the best of our knowledge, no published data specifically validating the drug uptake model of the HPS could be found. However, drug uptake behavior was found to be reasonable for a healthy patient [22] and should, therefore, not pose a bottleneck for such simulations.

The limitation of a simulator-based study is that simulation testing cannot mimic real-world performance under clinical conditions in all its extent. A further limitation could also be that performed this study on a single anesthesia workstation. However, using the experimental conditions as described above in a controlled setting (using the simulator), we have found that the effect of the reservoir bag disconnection may be clinically relevant, if not severely dangerous to the patient. To reduce potentially confounding patient variables, we also used a human patient simulator as in the study by Luria et al. [22]. In the present study, we also noted that simulation using high-fidelity simulator manikins is a valid tool for training users to understand the proper functioning of the anesthesia workstation and adverse events. The high-fidelity simulation was more widely adopted in medicine and is commonly used in the emergency and anesthetic specialties in which high-risk skill assessments are used more frequently [23]. Finally, it is important to be noted that the reported simulator-based setup can be used without potential clinical risks for patients in real settings. It was shown to be effective in evaluating the influence and severity of scenarios, similar to the one reported in this study.

## Conclusions

This simulator-based study demonstrates that reservoir bag disconnection leads to a significant decrease of inspired anesthetic gases and oxygen, because of entrainment of room air due to a large circle breathing system leak. During the disconnection of the anesthesia reservoir bag, the process of pharmacokinetics takes place faster compared to the classical wash-in and wash-out pharmacokinetic properties in the circle breathing system. The time constant was affected by the FGF rate, as well as by the gradient of the anesthetic gases between the circle and atmospheric air.

## Data Availability

The dataset generated and analyzed during the current study is available from the corresponding author on reasonable request.

## Abbreviations

F_circ_: Circuit fraction of gas
F_del_: Delivered fraction of gas
FGF: Fresh gas flow
F_I_N_2_O: Inspired fraction of N_2_O concentration
F_I_Sevo: Inspired sevoflurane concentration
F_I_O_2_: Inspired fraction of O_2_ concentration
HPS: Human Patient Simulator
N_2_O: Nitrous oxide
O_2_: Medical oxygen
PEEP: Positive end-expiratory pressure
*τ*: Time constant
V_D_: Amount of anesthetic agent delivered into the breathing system
${\dot{\mathrm{V}}}_D$: Rate of anesthetic agent delivered into the breathing system
VS: System volume
V_T_: Tidal volume
V_tot_: The total gas volume
${\dot{\mathrm{V}}}_{uptake}$: Rate of gas uptake
